# The impact of exercise on growth factors in postmenopausal women: a systematic review and meta-analysis

**DOI:** 10.1186/s12905-024-03240-7

**Published:** 2024-07-10

**Authors:** Yasaman Nasir, Mohammad Reza Hoseinipouya, Hesam Eshaghi, Mohammad Hossein Rahimi

**Affiliations:** 1 Department of Community Nutrition, School of Nutritional Sciences and Dietetics, Tehran University of Medical Sciences, Poorsina Street, Enghelab Avenue, Tehran, Iran; 2 Tehran Disaster Mitigation and Management Organization, Tehran, Iran; 3 Students’ Scientific Research Center (SSRC), Tehran University of Medical Sciences (TUMS), Tehran, Iran

**Keywords:** IGF-1, Insulin-like growth factor-1, Insulin-like growth factor binding proteins, IGFBP3, Postmenopause, Exercise, Meta-analysis

## Abstract

**Background:**

Aging results in many changes in health status, body composition, muscle strength, and, ultimately, functional capacity. These changes coincide with significant alterations in the endocrine system, such as insulin-like growth factor-1 (IGF-1) and IGF-binding proteins (IGFBPs), and may be associated with many symptoms of aging. The objectives of this study is to investigate the potential influence of different types of exercise, such as resistance training and aerobic training, on IGF-1 and IGFBP-3 levels in postmenopausal women.

**Methods:**

Medline, Scopus, and Google Scholar databases were systematically searched up to November 2023. The Cochrane Collaboration tool was used to assess the risk of bias and the quality of the studies. The random-effects model, weighted mean difference (WMD), and 95% confidence interval (CI) were used to estimate the overall effect. Between-study heterogeneity was assessed using the chi-squared and I^2^ tests.

**Results:**

Seventeen studies were included in the present systematic review and 16 studies were included in the meta-analysis. The pooled results from 16 studies (21 trials) with 1170 participants examining the impact of exercise on IGF-1 concentration showed a significant increase in IGF-1, and the pooled results among six studies (trials) showed a significant decrease in IGFBP-3 concentration (730 participants). In addition, resistance training and aerobic training had a significant effect on increasing IGF-1 concentration post-exercise compared with placebo.

**Conclusion:**

Based on this meta-analysis, Women who have completed menopause and followed an exercise routine showed changes in IGF-1 and IGFBP-3 levels that can indirectly be associated with risk of chronic age-related conditions.

## Introduction

IGF-1 (Insulin-like Growth Factor 1) is a hormone crucial for cell growth and development in the body. Its levels are linked to both high risks of cancer and accelerated aging with elevated levels, and decreased muscle mass and bone density with low levels. In post-menopausal women, changes in estrogen levels during menopause may affect IGF-1 levels, impacting metabolism, body composition, and overall health [1, 2]. In post-menopausal women, there is evidence to suggest that the decline in estrogen levels may be related to changes in IGF-1 levels. During menopause, estrogen production decreases, which can lead to changes in IGF-1 signaling and function. This hormonal shift may contribute to age-related changes in metabolism, body composition, and overall health in post-menopausal women [3].

One of the most essential and straightforward steps that individuals can take is to engage in regular exercise. The benefits of exercise during menopause include enhancements in cardiorespiratory function, offsetting metabolic risks related to declining estrogen levels, weight management, and bone density preservation, reduction of low back pain, stress reduction, mood improvement, and possible alleviation of hot flashes [4, 5]. It is emphasized that it is never too late to start exercising, and individuals should begin gradually with activities they enjoy, such as walking, cycling, strength training, swimming, or attending fitness classes. Consistent physical activity, even at a moderate level, can significantly contribute to overall well-being and energy levels [6].

Consequently, many researchers are seeking the efficacy and safety of non-pharmacological interventions to optimize IGF-1 levels and to offer methods for disease prevention in postmenopausal women. The level of IGF-1 can indirectly be associated with the development of certain diseases. Studies have shown that low IGF-1 levels may be linked to an increased risk of chronic age-related conditions such as neurodegenerative disorders, Alzheimer’s, diabetes, cardiovascular diseases, and overall decline in strength and immune function. Furthermore, elevated levels of IGF-1 may be associated with certain diseases such as various types of cancers, certain cardiovascular diseases, and metabolic disorders.

In this regard, modification of habitual lifestyle factors has been proposed as a means of influencing IGF-1 levels. Some clinical trials have shown that increased physical activity (PA) advantageous in this field [7].

The novelty of this study stems from its comprehensive approach to systematically reviewing the existing literature, assessing methodological quality and bias, and conducting a quantitative analysis through meta-analysis. By investigating the effects of different types of exercise, such as resistance training and aerobic training, on IGF-1 and IGFBP-3 concentrations, this study aims to provide valuable insights into the potential mechanisms through which PA may influence these growth factors in postmenopausal women.

Furthermore, the unique aspect of this study is the direct comparison of the effects of PA on IGF-1 and IGFBP-3 levels in postmenopausal women compared to control groups. This comparative analysis adds a novel dimension to the existing knowledge base and offers a more comprehensive understanding of the impact of exercise on these crucial biomarkers in the context of postmenopausal health. Furthermore, previous research has shown inconsistencies in the effects of PA on IGF-1 levels [7, 8], with some studies reporting no changes or even a decrease in IGF-1 levels following exercise. This inconsistency underscores the need for a systematic review and meta-analysis to synthesize the existing evidence and provide more clarity on the impact of exercise on IGF-1 and IGFBP-3 concentrations in postmenopausal women.

Therefore, this study aimed to: (1) systematically review the existing literature on the impact of exercise on insulin-like growth factor-1 (IGF-1) and insulin-like growth factor-binding proteins (IGFBPs) in postmenopausal women. (2) To assess the methodological quality and risk of bias in the identified studies through the utilization of the Cochrane Collaboration tool. (3) To quantitatively analyze the effects of exercise on IGF-1 and IGFBP-3 concentrations in postmenopausal women through a meta-analysis of relevant studies. (4) To investigate the potential influence of different types of exercise, such as resistance training and aerobic training, on IGF-1 and IGFBP-3 levels in postmenopausal women.

## Methods

### Search strategy

**Fig. 1:**
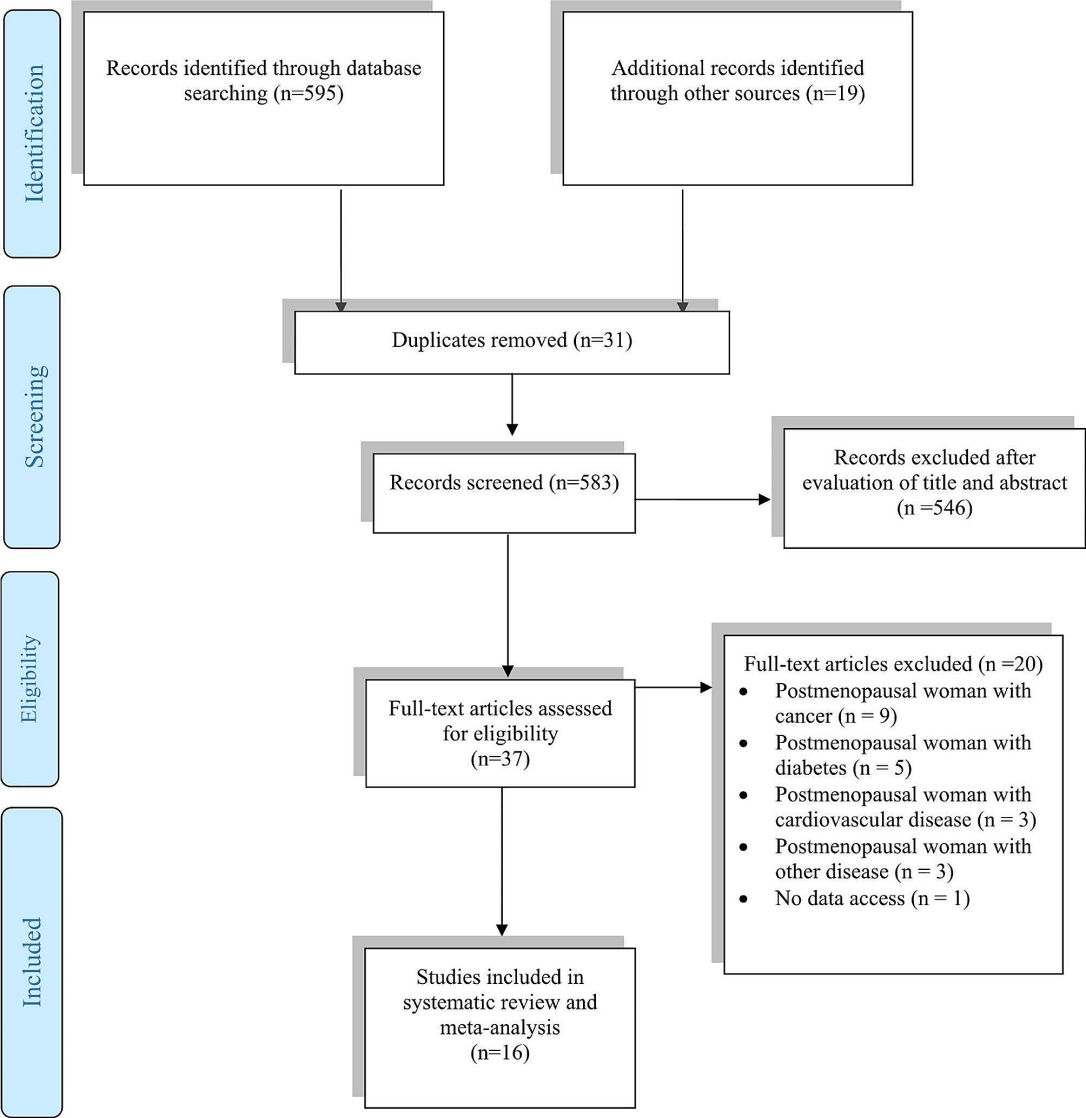
Preferred reporting items for systematic reviews and meta-analyses (PRISMA) flow diagram of study selection process

This systematic review and meta-analysis was performed according to the Preferred Reporting Items for Systematic Reviews and Meta-Analyses (PRISMA) [9]. A computerized literature search was performed from inception to November 2023 using Medline, Scopus, the Cochrane Library, and Google Scholar. The following phrases and their combination headings, (MeSH) and non-MeSH terms, were used: “IGF-1”, “insulin-like growth factor-1”, “somatomedin”, “insulin-like growth factor binding proteins”, “IGFBP”, “IGF-binding proteins”, “IGFBP-3”, “somatomedin-binding proteins”, “menopause”, “post menopause”, “elderly”, “aging”, “middle aged”, “older women”, “postmenopausal period”, “exercise programs”, “weight-bearing strengthening program”, “strengthening program”, “exercise”, “exercise training”, “resistance exercise”, “resistance training”, “physical activity”, “controlled trial”, “randomized”, “randomised”, “random”, “randomly”,“ randomized clinical trial”, “ RCT”, “ blinded”, “ double blind”, “double blinded”, “ trial”, “ controlled clinical trial”, “crossover procedure”, “cross-over trial”,“ double blind procedure “and “equivalence trial”. The reference lists of all the articles were examined to identify eligible studies (Fig. 1).

### Eligibility criteria

Studies were selected by applying the following Population-Intervention-Comparator-Outcomes-Study (PICOS) design criteria [9]: (1) original randomized-controlled trial studies; (2) healthy subjects performed aerobic, resistance, or both training interventions as an exercise strategy; (3) at least one outcome measure of IGF-1 and IGFBP3 was reported; (4) presented data of interest as mean and standard deviation (SD) of IGF-1 and IGFBP3 in both the intervention and placebo groups; and (5) had a trial duration of more than 1 week. The exclusion criteria were as follows: (1) studies evaluating exercise in postmenopausal women with diseases such as cancer, diabetes, and cardiovascular disease; (2) semi-experimental, non-randomized trials and trials without control groups; (3) duplicate studies with the same population (several papers reported the same data); (4) experimental and animal studies; and (5) reviews, letters to editor, editorial articles, or case reports.

### Selection strategy

All recorded articles found from electronic or manual searches were entered into the Endnote software for screening (EndNote X6, Thomson Reuters, New York). After the primary search, titles and abstracts of the papers included in the search strategy were screened. The authors independently selected the studies according to the inclusion criteria. Articles that met the eligibility criteria for title and abstract screening were selected for full-text review. Parallel clinical trials using a control group were selected for systematic review and meta-analysis. All categorized trials were retrieved by either of the authors. On the basis of the full information, we used a standardized form to select trials eligible for inclusion in the review. Contradictions between the authors were resolved by consensus or by a third researcher.

### Data extraction

The following data was extracted: first author’s name, publication year and country, research design, age of subjects, sample size, duration of intervention, type of exercise, and training status using a pre-designed standardized electronic form (Excel, Microsoft Office). In addition, we extracted the mean and SD of IGF-1 and IGFBP3 levels at baseline and after the intervention.

Any reported standard error of the mean (SEM) was converted to an SD using the following formula: SD = SEM × √n (n is the number of participants in each group). Finally, in studies that reported data in graphical figures, data extraction was performed using the GetData Graph Digitizer 2.24 [10].

### Study quality

As it has been accepted that trial inclusion with a high risk of bias may distort the results of a meta-analysis [9, 11], the Cochrane Collaboration tool was used to assess the risk of bias. The quality of all included studies was evaluated for the following items: randomization sequence generation, allocation concealment, blinding of participants, personnel, investigator, and assessor, attrition rates, and financial interest by companies (Table 1). These were rated as having a low, unclear, or high risk of bias. An RCT was ranked as having low, medium, or high overall risk based on the key areas of allocation concealment, reporting of attrition rates, and participant and assessor blinding (high = all four factors rated high/medium = 2 or 3 factors rated high or unclear, and low = all key areas rated low).

**Table 1:** Cochrane risk of bias assessment

Study	Random Sequence Generation	Allocation concealment	Blinding of participants and personnel	Blinding of outcome assessment	Incomplete outcome data	Selective outcome reporting	Other sources of bias	Overall Risk of Bias
Yoon et al. (2019)	U	U	H	H	L	L	U	Medium
Son et al. (2019)	L	L	U	L	L	L	L	Low
Cunha et al. (2018)	L	U	U	U	L	L	L	Medium
P. Nunes et al. (2017)	U	U	H	H	L	L	U	Medium
Mason et al. (2013)	L	U	U	U	L	H	U	Medium
Ohta et al. (20,012)	U	U	H	H	L	L	L	Medium
Friedenreich et al. (2011)	L	U	U	U	L	L	U	Medium
Seo et al. (2010)	L	U	H	H	L	L	U	Medium
de Souza Vale et al. (2009)	U	U	H	H	L	L	U	Medium
Orsatti et al. (2008)	U	H	H	H	L	L	U	Medium
McTiernan et al. (2005)	L	U	H	H	H	H	U	Medium
Milliken et al. (2003)	U	H	H	H	L	L	U	Medium
Copeland et al. (2003)	U	U	H	H	L	L	L	Medium
Alev Ay et al. (2003)	U	U	U	U	L	L	L	Medium
Parkhouse et al. (1999)	U	H	H	H	L	H	U	High
Kohtr et al. (1995)	U	U	H	H	L	L	L	Medium

*L* low risk of bias; *H* high risk of bias; *M* medium risk of bias; *U* unclear risk of bias

### Analysis and measures of treatment effect

For every study, the mean changes and SDs were computed for continuous variables. For studies with no reported SD of the mean difference, the following formula was used: SD change = square root [(SD baseline ^2^ + SD final ^2^) − (2 × 0.8 × SD baseline × SD final)] [12]. Standardized mean changes were used for variables pooled on different scales. Between-study heterogeneity was assessed using the chi-square (χ2) test and quantified using the I^2^ statistic, which represents the percentage of total variation across studies that is ascribable to heterogeneity rather than chance. I^2^ was calculated using the formula I^2^ = 100% × (Q − df)/Q, and an I^2^ value of 75% or greater indicated a high level of inconsistency (Q is the χ2 statistic, and df is the degree of freedom). Significant heterogeneity was defined as a P-value of < 0.05.

Random- and fixed-effect models were applied to assimilate the overall effect, where evidence of statistical heterogeneity and homogeneity was observed, respectively. An influence analysis was performed to determine whether the results could have been distinctly affected by a single study [13].

To identify the possible sources of between-study heterogeneity, pre-planned subgroup analyses were performed based on the type of exercise (resistance training, aerobic training, and a combination of resistance and aerobic training), trial duration (months), baseline body mass index (BMI), baseline IGF-1and IGFBP-3 and mean age (≥ 60 years and < 60 years). Baseline IGF-1and IGFBP-3 cut-off points were 119 ng/mL and 1.89 µg/ml, respectively, based on sex and age, according to Friedrich et al. [14].

Publication bias was assessed using Begg’s rank correlation test and Egger’s regression asymmetry test. Statistical analysis was performed using the STATA 11.2 software (StataCorp, College Station, Texas, USA).

## Result

### Overview of included studies

We initially identified 614 papers from databases and internet searches and included 17 studies in the present systematic review. Sixteen studies were selected according to the 4-phase flow diagram shown in Fig. 1 in the present meta-analysis. The characteristics of the included studies are summarized in Table 2, which provides an overview of the methodological quality of each selected trial. The included studies were those with a number of subjects ranging from eight to 308, most of which used an RCT design. The participants tended to be older, with a mean age of 56.4, and all were postmenopausal women (*n* = 1170). In the 16 publications included in this study on IGF-1, the total participant pool for IGF-1 was 1170 and for IGFBP-3 was 730 in six publications. One study [15] only reported baseline data, and we did not receive raw after-trial data from the authors. This number includes people who dropped out of some studies.

**Table 2:** Summary of relevant sources of data included for meta-analysis

Author	Study Design Characteristics							Blinding Status		Sample Size		IGF-1 Status	IGFBP-3 Status
	Design	Training status	Training	Exercise type #	baseline BMI (kg/m^2^)	Mean age (Y)	Duration (M)	Participant	Personnel	Exercise	Control		
Yoon et al. (2019)	RCT	U	resistance and interval training	1	22.5	64	2.8	Unblined	Unblined	10	10	↑	
Yoon et al. (2019)	RCT	U	resistance and aerobic exercise	1	22.5	64	2.8	Unblined	Unblined	10	10	↑	
Son et al. (2019)	RCT	U	resistance training	2	26.6	67.5	2.8	Unblined	Blinded	12	13	↑	
Cunha et al. (2018)	RCT	U	single-set resistance training	2	27.1	68.8	2.8	Unblined	Blinded	20	21	↑	
Cunha et al. (2018)	RCT	U	multiple-sets resistance training	2	27.1	68.8	2.8	Unblined	Blinded	21	21	↑	
P. Nunes et al. (2017)	RCT	U	low volume resistance training	2	28.8	60.9	3.7	Unblined	Unblined	10	12	≡	
P. Nunes et al. (2017)	RCT	U	high volume resistance training	2	28.8	60.9	3.7	Unblined	Unblined	12	12	≡	
Mason et al. (2013)	RCT	U	aerobic exercise	3	27.8	57.5	12	Unblined	Blinded	117	87	≡	≡
Ohta et al. (20,012)	RCT	U	aerobic exercise	3	22.6	71.8	2.8	Unblined	Unblined	13	13	≡	≡
Friedenreich et al. (2011)	RCT	U	aerobic exercise	3	29.1	60.9	6 & 12	Unblined	Blinded	154	154	≡	≡
Seo et al. (2010)	RCT	U	aerobic exercise	3	25.1	55.6	2.8	Unblined	Unblined	7	7	≡	
Seo et al. (2010)	RCT	U	combined walking and resistance training	1	25.1	55.6	2.8	Unblined	Unblined	8	7	≡	
de Souza Vale et al. (2009)	RCT	U	aerobic exercise	3	28.5	68.55	3	Unblined	Unblined	13	10	↑	
de Souza Vale et al. (2009)	RCT	U	strength training	2	28.5	68.55	3	Unblined	Unblined	12	10	↑	
Orsatti et al. (2008)	RCT	U	resistance training	2	28.2	58.5	13.7	Unblined	Unblined	21	22	↑	
McTiernan et al. (2005)	RCT	U	aerobic exercise	3	30.4	60.6	3 & 12	Unblined	Unblined	87	86	≡	≡
Milliken et al. (2003)	RCT	U	resistance and weight-bearing exercises	1	25.9	55.6	6 & 12	Unblined	Unblined	26	30	≡	≡
Copeland et al. (2003)	RCT	U	resistance training	2	28.65	54.2	0.9 & 3	Unblined	Unblined	8	8	↑	
Figueroa et al. (2003) *	RCT	U	resistance and aerobic exercise	1	25.7	57.6	12	Unblined	Unblined	20	26	**^**	
Alev Ay et al. (2003)	RCT	U	aquatic aerobic exercises	3	29.2	54.6	6	Unblined	Blind	21	20	↑	
Parkhouse et al. (1999)	RCT	U	resistance training	2	26.5	68	8	Unblined	Unblined	13	9	↑	≡
Kohtr et al. (1995)	RCT	U	weight-bearing exercise training	3	24.4	65.5	11	Unblined	Unblined	8	8	≡	

*RCT* Ran domized controlled trial; *BMI* Body mass index; M = month; Y = years; T = trained; U = untrained

**^**Unspecified or unknown. ↑ Exercise group significantly higher compare to control group; ≡ No significant difference between trials

*Excluded from meta-analysis; # Exercise type 1 = Aerobic & Resistance combination; 2 = Resistance; 3 = Aerobic

### Findings from the systematic review

The number of studies that demonstrated a significant effect of exercise on IGF-1 levels was equal to the number of studies that showed no effect. In addition, no study has demonstrated a significant effect of exercise on IGFBP-3. Yoon et al. [16] suggested that a combination of resistance and aerobic training for 12 weeks increased IGF-1 concentrations compared to the control group. These results are consistent with those of de Souza Vale et al. [17], who used a combination of resistance and aerobic training for 3 months, which increased IGF-1 concentration after exercise in comparison with placebo. Seo et al. and Milliken et al. studied the combination of resistance and aerobic training for 6 and 12 weeks and observed no significant increase in IGF-1 concentration compared with the control group [18, 19].

Most studies have reported the effects of resistance and aerobic training on IGF-1 levels. Additionally, the number of studies that investigated the effects of resistance training was equivalent to the number of studies that showed an aerobic training effect. However, most studies that investigated the effect of resistance training showed a significant effect of increasing IGF-1 concentration [20–24] except that of Nunes et al. [25] which showed no effect of 16 weeks of resistance training on IGF-1 concentration. In contrast to resistance training, most studies that investigated the effect of aerobic training showed no significant effect on IGF-1 concentration [26–30] except for that of Alev Ay et al. [31] which showed a significant effect of 6 months of aquatic aerobic exercises on increasing IGF-1 concentration.

### Results from quality assessments

The quality of bias assessment is shown in Table 1. Briefly, participants’ random allocation was mentioned in all the included trials. However, six trials described the method of random sequence generation [18, 21, 28, 29, 32, 33]. One trial reported on allocation concealment [20]. Most studies had a low risk of bias based on selective reporting; however, three studies had a high risk of bias [24, 29, 33]. All studies had a low risk of bias resulting from incomplete outcome data except one [29]. In addition, all studies had a high or unclear risk of bias as a result of blinding of participants and personnel and blinding of outcome assessments, except for one study that had a low risk of bias as a result of blinding of outcome assessment [32]. All studies had a low or unclear risk of bias regarding other potential threats to their validity.

### Findings from the meta-analysis

#### The effects of exercise on IGF-1 concentration

**Fig. 2:**
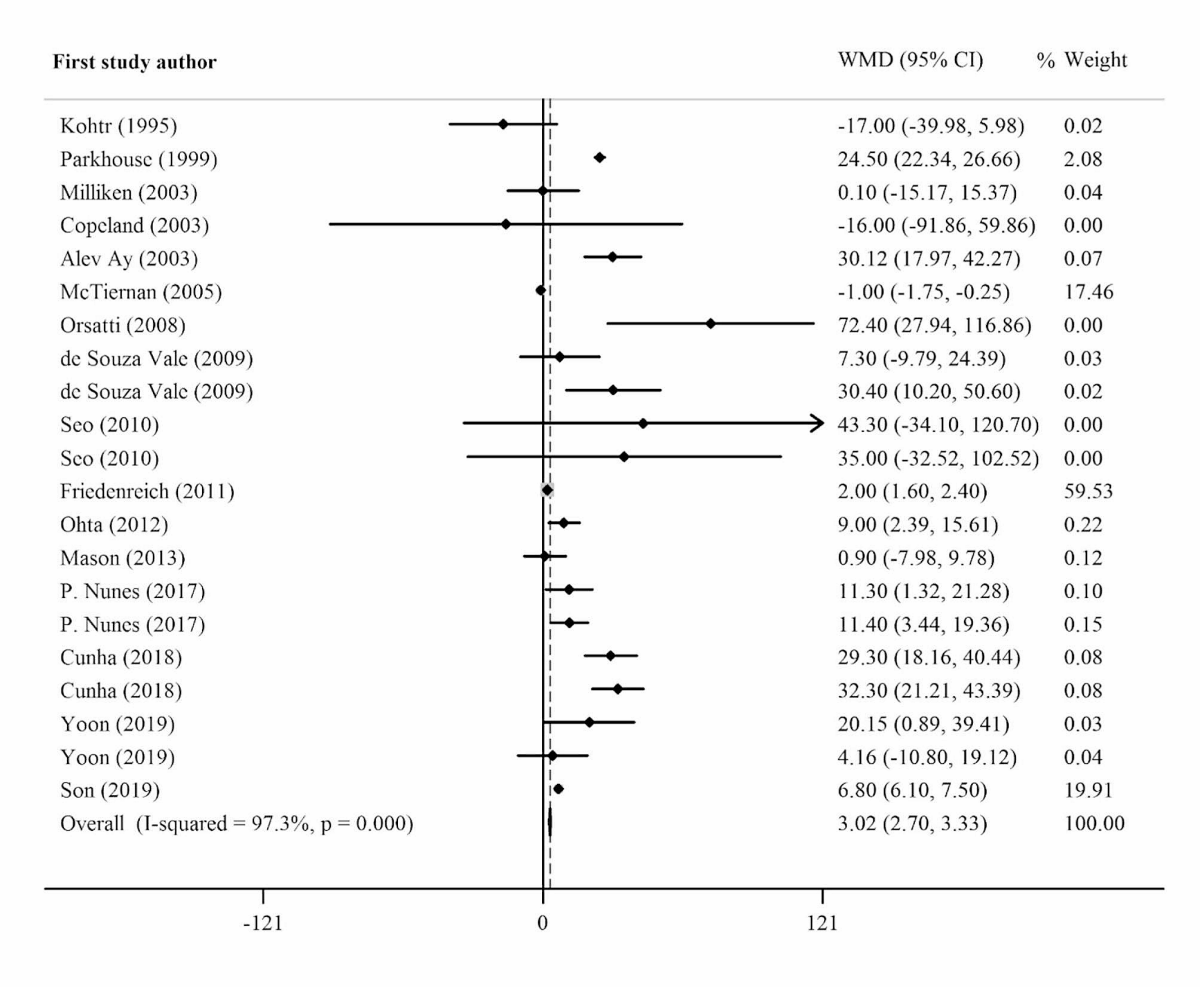
Forest plot of the effect of exercise on IGF-1 concentration, *WMD* Weighted mean difference; *CI* Confidence interval

The pooled results from 16 studies (21 trials) with 1170 participants that examined the impact of exercise on IGF-1 levels are presented in Fig. 2. Data included the weighted mean difference (WMD) and 95% confidence intervals (CIs). It is important to consider the overall relevance of these findings, particularly the significant increase in IGF-1 level. The positive effects of exercise could cause an increase in IGF-1 concentration regardless of the type of exercise (overall WMD = 3.02 ng/ml; 95% CI: 2.70 to 3.33); however, there was significant heterogeneity between studies (I^2^ = 97.2%; *p* = 0.00). In the subgroup analysis based on exercise type, we did not observe significantly higher values of IGF-1 concentration after a resistance and aerobic training combination (WMD = 7.25 ng/ml; 95% CI: -2.83 to 17.34) than in the placebo group, and there was no significant heterogeneity between studies (I^2^ = 11.1%; *p* = 0.337). In addition, Table 3 shows that resistance training had a significant effect on increasing IGF-1 concentration post-exercise compared with placebo (WMD = 21.35 ng/ml; 95% CI, 11.79 to 30.91). In addition, aerobic training had a significant effect on increasing IGF-1 concentration post-exercise compared to placebo (WMD = 3.13 ng/ml; 95% CI, 0.15 to 6.11). However, there was evidence of significant heterogeneity between the effect sizes of the included studies (*I*^2^ = 97.1%, *p* = 0.00; *I*^2^ = 90.8%, *p* = 0.00).

**Table 3:** Subgroup analysis to assess the effect of exercise on IGF-1 concentration

Subgrouped by	No. of trials	Effect size^1^	95% CI	*P* Value	I^2^ (%)	*p* for heterogeneity	
Exercise Type							
Combined resistance and aerobic training	4	7.255	-2.831 to 17.341	0.159	11.1	0.337	
Resistance training		9	21.353	11.795 to 30.911	< 0.001	97.1	< 0.001
Aerobic training	8	3.132	0.149 to 6.116	0.040	90.8	< 0.001	
**Trial Duration**							
< 3 months	11	16.714	8.946 to 24.482	< 0.001	77.8	< 0.001	
3–6 months	4	21.282	7.696 to 34.868	0.002	77.8	0.004	
≥ 6 months	6	7.054	1.008 to 11.117	0.021	99.0	< 0.001	
**Mean Age**							
≥ 60 years	14	11.518	7.708 to 15.328	< 0.001	98.1	< 0.001	
< 60 years	7	18.702	0.238 to 37.166	0.047	76.4	< 0.001	
**Baseline BMI**							
≤ 25 kg/m^2^	6	7.343	-2.489 to 17.174	0.143	36.1	0.166	
> 25 kg/m^2^	15	12.606	8.745 to 16.467	< 0.001	98.0	< 0.001	
**Baseline IGF-1**							
≤ 119 ng/ml	10	14.369	9.159 to 19.579	< 0.001	98.4	< 0.001	
> 119 ng/ml	11	8.143	3.462 to 12.824	0.001	40.1	0.082	

^1^Calculated by random effects model

*CI* Confidence interval

Furthermore, exercise increased IGF-1 levels in all other subgroups, except in RCTs that included women with BMI ≤ 25 kg/m^2^, with no considerable between-study heterogeneity (*I*^2^ = 36.1%; *p* = 0.16).

Interestingly, increased IGF-1 levels after PA in RCTs including women with higher baseline IGF-1 levels were lower than those in women with lower baseline IGF-1 levels (Table 3) without evidence of heterogeneity (*I*^2^ = 40.1%; *p* = 0.082).

#### Effects of exercise on IGFBP-3 concentration

**Fig. 3:**
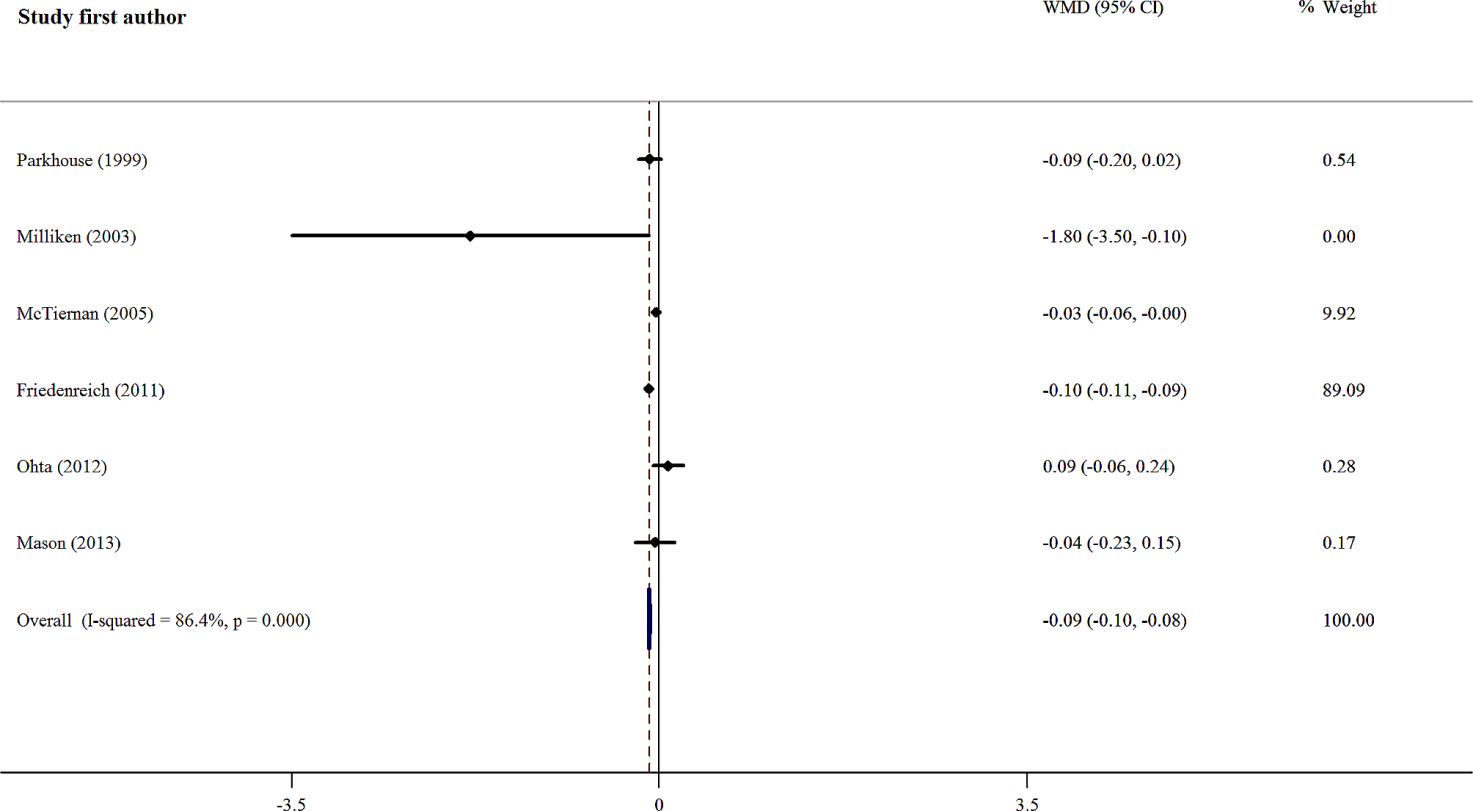
Forest plot of the effect of exercise on IGFBP-3 concentration, *WMD* Weighted mean difference; *CI* Confidence interval

Pooled results among six studies (trials) that recorded IGFBP-3 concentration (730 participants) showed significant differences in IGFBP-3 concentration post-exercise (overall WMD = -0.09 µg/ml; 95% CI, -0.10 to -0.08), but there was significant heterogeneity between studies (Fig. 3). We did not find a source of heterogeneity for IGFBP-3; however, the subgroup analyses are presented in Table 4. In this subgroup, 4 trials used aerobic training [28, 29, 33, 34], one trial used a combination of resistance and aerobic training [19] and another study used resistance training [24]. We did not observe significantly lower IGFBP-3 concentrations after aerobic training (WMD = -0.04 µg/ml; 95% CI, -0.11 to 0.00) compared to placebo in 4 trials using aerobic training. There was significant heterogeneity between studies (*I*^2^ = 86.4%; *p* = 0.00) (Table 4).

**Table 4:** Subgroup analysis to assess the effect of exercise on IGFBP-3 concentration

Subgrouped by	No. of trials	Effect size^1^	95% CI	*P* Value	I^2^ (%)	*p* for heterogeneity
Exercise type						
Combined resistance and aerobic training	1	-1.80	-3.50 to -0.10	0.038	-	-
Resistance training	1	-0.09	-0.20 to 0.02	0.099	-	-
Aerobic training	4	-0.04	-0.11 to 0.00	0.169	86.4	0.000
**Mean Age**						
≥ 60 years	4	-0.71	-2.38 to 0.97	0.407	75.3	0.044
< 60 years	2	-0.05	-0.11 to 0.01	0.080	90.8	< 0.001
**Baseline IGFBP-3**						
≤ 1.89 µg/ml	1	-1.800	-3.505 to -0.095	0.038	-	-
> 1.89 µg/ml	5	-0.053	-0.109 to 0.004	0.068	87.8	< 0.001

^1^Calculated by random effects model

*CI* Confidence interval

**Fig. 4:**
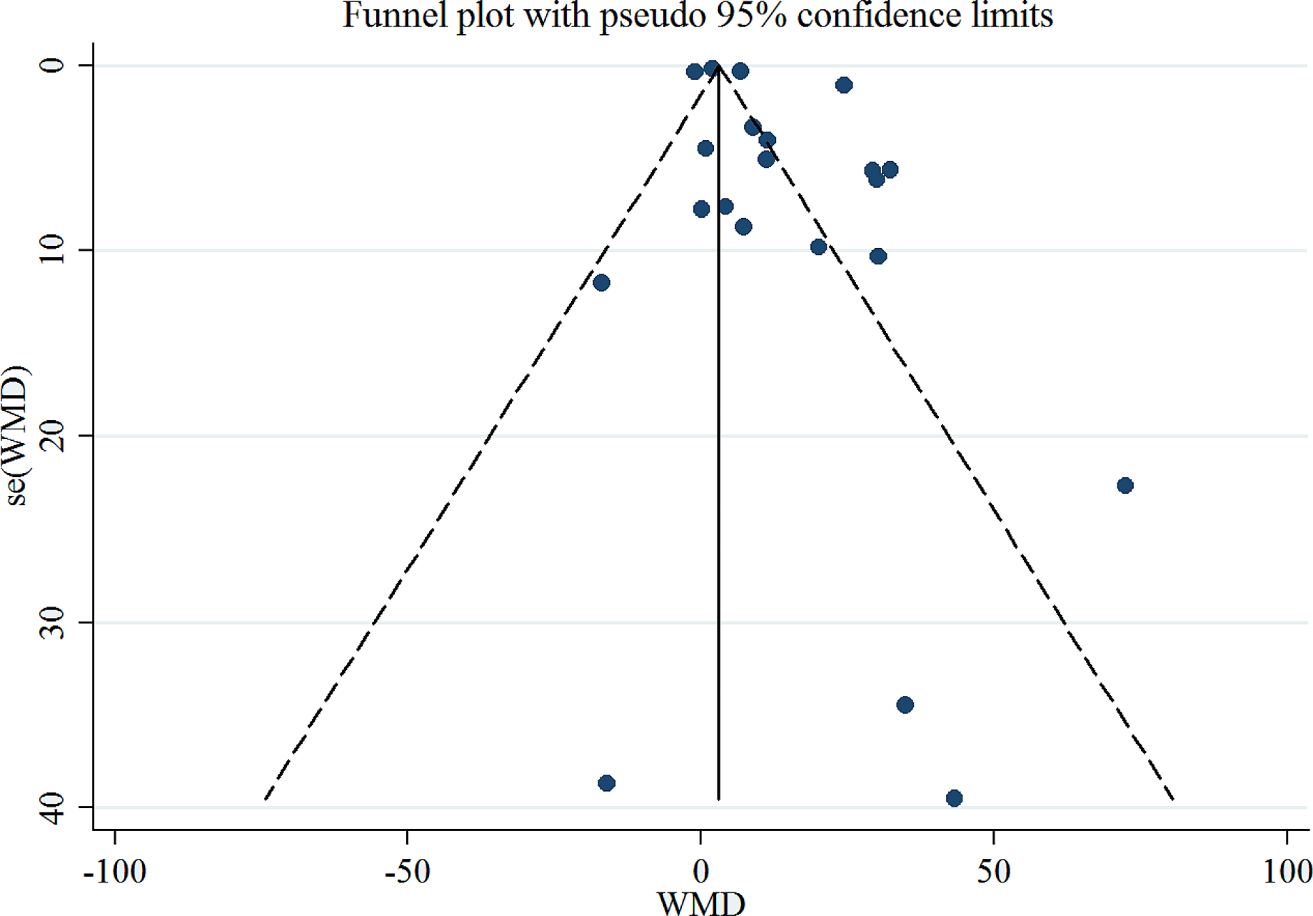
Funnel plot for evaluating publication bias in IGF-1

***Sensitivity analysis and small‑study effects*** We re-analyzed the data to identify the overall influence of a single study. Sensitivity analysis indicated that the results were not significantly influenced by any study on IGF-1 and IGFBP-3 concentrations. Visual inspection of the funnel plots revealed evidence of asymmetry for IGF-1 (Fig. 4); however, there was no evidence of publication bias among the studies examining the effects of exercise on IGF-1 concentration (*p* = 0.469, Begg’s test). Furthermore, there was no evidence of publication bias among the studies after examining the effects of exercise on IGFBP-3 levels (*P* = 0.51, Egger’s test).

## Discussion

There is evidence that maintaining physiological function and metabolism in postmenopausal women is linked to plasma IGF-1 and IGFBP-3 levels [4, 35, 36].

In 2017, a meta-analysis of seven studies was performed to evaluate the effect of programmed exercise on IGF-1 and IGFBP-3 levels in postmenopausal women. No significant differences were seen between the mid- and long-term exercise intervention versus controls in IGF-1 and IGFBP-3 levels [37]. In 2016, a meta-analysis of 5 studies assessed the effects of PA in breast cancer participants, most of whom were postmenopausal women. PA has been reported to be an effective intervention for the reduction and induction of IGF-1 and IGFBP-3 levels [38].

Since the meta-analyses mentioned above provide a snapshot of knowledge at the time of incorporating data from studies identified during the latest search, newly identified studies can change the conclusions of those reviews. Therefore, we aimed to include all RTCs to summarize the current findings in this field.

The present meta-analysis of randomized trials indicated that PA significantly increased and decreased IGF-1 and IGFBP-3 levels, respectively. Subgroup analysis showed that resistance and aerobic training significantly increased IGF-1 levels. Furthermore, it did appear that PA significantly increased IGF-1 levels in women with a BMI of > 25 kg/m^2^. Current evidence suggests that IGF-1 levels are associated with BMI whereby they are negatively related to obesity [39]. In contrast, PA can optimize IGF-1 levels under both elevated and reduced conditions [40]. Therefore, our results are reasonable because PA led to increased IGF-1 levels in overweight and obese participants. We also showed that IGF-1 levels were higher in women with a higher baseline IGF-1 level than in those with a lower baseline. Similar conclusions have been reported in a previous study, which reported that baseline IGF-1 level could be a predictive marker of the response to PA [40].

The mechanism by which PA affects IGF-1 and IGFBP-3 levels has not been fully elucidated. Some studies have proposed that systemic IGF-1 levels may change due to autocrine alterations in exercise-responsive tissues, including adipocytes and skeletal myocytes, or alterations in the expression of IGF-1 receptors (IGF-1R) [40]. IGFBP-3 as a buffer could show an adaptive response to control metabolic reactions [41]. However, the effects of PA on IGF-1 and IGFBP-3 levels largely depend on several factors. Postmenopausal hormone therapy is an important factor. Steroid hormones affect the secretion of hepatic IGF-1 and have various effects on IGF-1 level [42]. Growth hormone, the principal inducing factor for IGF-I synthesis in the liver, is further stimulated by insulin. Therefore, these hormones could potentially contribute to changes in IGF-I level [41]. Medications, smoking, age, BMI, health status, diet, acute or chronic PA, trained/untrained condition of subjects [40, 43–45], and IGF-1 and IGFBP-3 gene polymorphisms in relation to circulating levels of these proteins should be considered [1]. The intra- and inter-approaches to variability in IGF-1 and IGFBP-3 measurements may also contribute to different clinical responses [46] and ultimately have repercussions for decisions in clinical practice.

The present meta-analysis had several limitations. No included studies in this meta-analysis blinded the providers/ assessors and only one study blinded the participants. Due to the nature of physical activity interventions, blinding in such studies may be challenging. Moreover, evidence was downgraded due to the lack of homogeneity among included articles and subgroup hypothesis were not sufficient for founding the source of heterogeneity.

### Side effects

No injuries, complications, or adverse effects were observed among the participants who completed the intervention protocols.

### Implications for practice

The present meta-analysis of randomized trials suggests that PA might be the easiest, simplest, and most accessible program for postmenopausal women to increase IGF-1 and IGFBP-3 levels. Both high and low circulating IGF-1 levels are associated with pathophysiological status [4, 35]. Malignant tissues are capable of overexpressing IGF-1R and producing IGF ligands in an autocrine or paracrine manner [40]. Therefore, PA should be considered in these subjects, although it may optimize the IGF axis in subjects with either elevated or reduced IGF-1 expression [40]. Our results cannot be generalized to those with other health presentations not included in this analysis, such as postmenopausal women with diabetes or cardiovascular disease.

### Implications for research

As mentioned previously, the blinding of PA interventions may be challenging. A recent meta-analysis reported that the possibility of blinding care providers/assessors/participants in PA interventions was poor. This could result in an underestimation of treatment effects [47]. Therefore, further studies are needed to develop high-quality and effective trials to translate recommendations into practice. Examining whether longer periods of PA intervention have sustained greater effects on changes in IGF-I and IGFBP-3 levels is needed. Effects of confounding factors on the IGF axis and PA, such as body composition, hormone therapy, IGF-1 and IGFBP-3 gene polymorphisms, and different dietary patterns should also be considered.

## Conclusion

After analyzing data from 16 randomized controlled trials, our findings suggest that implementing PA can serve as a straightforward and convenient method for postmenopausal women to potentially boost their IGF-1 and IGFBP-3 levels. However, the need for additional research is vital to solidify and expand upon these results, ensuring the validity and broader applicability of these findings in the future.

## Data Availability

Data supporting the findings of this study are available from the corresponding author upon reasonable request.
